# Root-Filling in Its Relation to Disease

**Published:** 1890-07

**Authors:** Rodrigues Ottolengui


ARTICLE VI.

### ROOT-FILLING IN ITS RELATION TO DISEASE.

BY RODRIGUES OTTOLENGUI, M. D. S.

[Read at the Anniversary Meeting of the Second District Society held at Newburgh, N. Y., June 9, 1890.

It has been recently charged that we of the Dental profession have no definite methods; and the diversity of opinion as to root-filling was cited as an evidence of the correctness of the statement. It is the purpose of this paper to show that there are different methods of filling roots because the conditions vary, to describe briefly some of the more constantly occurring phases, and to indicate in each the best method of filling the root.

It seems to me unfortunate that many men are filling all roots in one way—that they adopt a pet system and use it in all cases. The more observant dentist differentiates between differing conditions, and has chosen a method which seems best adapted to each.

The main points in dispute are:

1. Shall the canal be enlarged, or not?

2. Shall the root be dressed with medicament? and if so, with what?

3. Shall the root be filled at the first sitting?

4. Shall we rely on one filling-material for all cases? and if so, which shall it be?

To all of these queries the replies must be variable. The first three may be answered "Yes" and they may be answered "No." The last question is the one which is most disputed, mainly because few men have tried many materials; but the true answer, as in the other cases, is that the material must be dictated by the condition.

Thus, I cannot consider the subject in the order of these queries, but will rather choose to take it up in an orderly consideration of the different conditions in which teeth are presented to us, discussing each problem in its relation to each state of disturbance.

The first conditions, and the simplest, is where a patient presents with an aching pulp. There are three subdivisions of this class, each of which must receive notice if we are to be thorough in this discussion.

(A) The tooth has ached slightly, and it is decided that the pulp must be destroyed. Let us suppose that arsenic is applied, and that on the following day the pulp is removed entire, with slight pain. The procedure is simple. With the pulp before us we determine exactly the size of the canal. If it is sufficiently large to insure thorough filling, it need not be touched with a reamer, but should better be filled as it is. Immediate root-filling is indicated; delay is dangerous—the inviting home for bacteria should be filled at once. Shall a medicament be used? I think that a coagulant is here indicated, and that the one better than all others is chloride of zinc. Chloride of zinc is also a circumscribed caustic. On general principles, I should say not only that a coagulant is not useful in a pulpless tooth, but that it is actually harmful and therefore contra-indicated. Therefore the statement that it is indicated in a tooth from which a pulp has recently been removed entire needs explanation and is open to argument. Whether we consider the contents of the canaliculi of dentine to be neural tissue, or merely a lower order of animal matter which has the power of conveying impressions to the pulp, we are all agreed that they receive their life impulse from the pulp and not from the pericementum. This is shown by the absence of sensation in pulpless teeth, and further by the occasional discoloration of such teeth. This, then, is my reason for suggesting chloride of zinc in the class of teeth under consideration. The pulp being removed, and the medicament applied, what results? The escharotic property is exerted at the point immediately in contact, and the animal matter is destroyed. But this action is circumscribed. Somewhat deeper, a coagulum is formed. But there is another property which that agent possesses, and that is the *mumification* of *animal tissues*. This, I conceive, is what occurs in the minutest portions of the tubuli. Experience has shown me that teeth thus treated do not discolor to an objectionable extent.

(B) Condition similar to last, but, upon endeavoring to remove the pulp, excessive hemorrhage is produced. It becomes impossible to remove the pulp except in shreds, because of the tenacious attachment of the odontoblastic layer to the canal walls. In the first condition I said that if the size of the pulp as seen indicated that the canal was large, a reamer is not to be used. This implied that if the canal were small, it should be enlarged. But it is different in this second condition. Not only are we in doubt as to whether we have removed all of the pulp tissue, but we are reasonably sure that odontoblasts are still clinging to the canal walls. A reamer is the only sure way to become satisfied that the canal is thoroughly cleansed. If used, it will be found that at first the debris is stained with blood; but later it is found to be pure white—a satisfactory evidence that all is thoroughly accomplished. At this point, of course, we are to consider the use of a medicament. If the hemorrhage ceases, the procedure is the same as in the last instance—a dressing of dilute chloride of zinc for a few moments, followed by immediate root-filling. If the hemorrhage is uncontrollable, a dressing of equal parts of oil of cloves and creosote may be used, and the tooth temporarily filled. If the patient can return in two or three hours, it would be well to fill the root at such a time. It should be done as soon as possible.

(C) I come now to a condition which I have never seen explained, though one which I have met frequently. Patient presents with aching tooth. Diagnosis pulpitis, although no actual exposure is to be found. Arsenic is applied. Patient returns in a few hours in increased agony. Dressing is removed, and in succession all the drugs in the office are used to relieve the pain, but are ineffectual, till finally the pain becomes more bearable, lessened by the time which has elapsed since the removal of the arsenic. Patient is dismissed with some soothing agent in the cavity. Reports next day that a most uncomfortable night has been passed. Tooth found to be less sensitive to the touch, and decay is thoroughly removed. Approaching the pulp, however, evidence of vitality is found, and arsenic is cautiously applied in small portion. The experience of the day previous is repeated, and recurs for several days. In a case of this character the patient suffers greatly from pain and exhaustion from loss of sleep, and the operator is amazed to find arsenic so inefficient. Perhaps he buys a new supply. The diagnosis is simple if the symptoms are exactly as described, and the method of procedure the only one which will save the patient several days of torture. In these cases the pulp has begun to calcify. Frequently the entrance to the chamber is filled with pulp stones. These form an effectual barrier to the full activity of the arsenic, but sufficient enters between the globular masses to produce the great disturbance which results. As soon as the patient returns the first time complaining that the pain is unendurable, and it is found the soothing agents do not give relief, an anæsthetic should be administered and the pulp stones and the pulp itself removed while the patient is unconscious. The canal should be reamed out as thoroughly as possible, and the root filled at once. The coagulant is contra-indicated here, because nature has already cared for the contents of the tubuli. They are in a condition which will not change from the removal of the pulp; and the only change of color which will occur in the tooth is that dense and somewhat oily appearance by which we always detect teeth which have calcified pulps.

Having described these three subdivisions of the first class of teeth, we may now discuss filling-materials and the method of filling. It has been charged against a reamer in a root canal, that it is manifestly impossible to ream around a curve. This is not entirely true. Where the canal is tortuous, there being a curve along its main length, the flexible reamer, which has a safe end, in the hands of the expert, converts the passage into a straight canal by removing the curve walls. When there is a sharp angle at the extremity, the safe end of the reamer bars farther progress and guards against drilling through the tooth at that point, which not only would frequently result in disastrous consequences, but deceives the operator into believing that he has merely passed through the foramen, whereas in fact he has not reached the foramen at all, but leaves uncleansed that curved portion. Having reamed a root which has a sharp angle at its extremity as far as the safe point will permit, an exploration is made with a flexible unbarbed broach, and on removal the presence of this curve is indicated by the bend in the broach. This being known, the canal is reamed even larger if possible, to allow free approach to this danger point. Then Dr. Evans's silver-pointed root-dryer is heated and used, the hot fine point being readily forced into this curvature, so destroying any pulp tissue which may be left at that point, and by searing the walls sufficiently enlarges the canal at its extremity for the ingress of the filling-material to be used. In these cases I use a thin oxy-phosphate pumped in with a fine instrument till I am satisfied that the walls are smeared thoroughly. I then complete the filling by sliding into the mass a cone of gutta-percha. My object is this: The cone serves a double purpose; it forces the soft oxy-phosphates ahead of it, and it renders the root-filling more easy of removal should future exigencies make this necessary, as, should the crown be lost and an artificial substitute be needed. We should never fill roots with any material which cannot be removed. This is why wood, which some use, is especially to be avoided. Wood cannot be pulled out, and it is difficult to be drilled out.

The second condition to be considered is where the patient has allowed the pulp to die, and shortly after presents in great pain, suffering with pericementitis. This is familiar to all of us. The tooth is sore to the touch, the patient wants relief, and yet does not wish to submit to an operation of any kind. I sometimes give an anæsthetic and thoroughly open the entrance to the canal. Occasionally it will be sufficient to tie a silk ligature around the neck of the tooth and allow the patient or assistant to pull on the tooth while the engine work is being accomplished. Access being obtained to the chamber, it remains to remove the dead and putrescent pulp. Nothing which comes to us is more difficult to accomplish without pain to the patient or, by pushing a portion of this dead material beyond the apex, causing an infection which will result in abscess. Peroxide of hydrogen is useful in this place, but must be handled with extreme caution. In the first place, be sure that the agent is neutral. It is difficult to keep pure, and many manufacturers add a little sulphuric acid to it for the purpose of preserving it. This may be detected with test papers or other chemical tests for acids. If you are sure that you have a pure article, it may be used with a syringe, a few drops being forced into the canal, the needle not being pressed in to any depth. Allow a few moments to elapse, that the whole mass may become saturated by capillary attraction. Then remove the dead pulp as may be most readily done, the circumstances indicating the method. At this initial sitting—the tooth being quite sore—a reamer should not be used, but a paliative dressing of one of the essential oils should be gently placed in the root and covered with a temporary stopping. Should the tooth be elongated to an extent which makes it painful to close the mouth, the following method of procedure is attended with the happiest results: Take an impression of the affected tooth and that on each side of it, using 24k. gold, about 34g.; swage up a splint (three continuous crowns) which will cover the three teeth. This splint is fastened in place with cement, and a hole drilled through corresponding with the cavity in the tooth under treatment. This effectually prevents any pressure from being brought to bear on the tooth in occlusion, and does not interfere with the subsequent dressings. These dressings must be renewed daily until all soreness has subsided. They are to be preferably of the essential oils, no coagulant of any kind being under any consideration placed in the canal. It may be as well to mention here some of those agents which may and which may not be used. I copy from the list published by Dr. Harlan: The oils of cloves, cinnamon, cassia, turpentine, sassafras, camphor, cajeput, sanitas, boracic acid, chloroform, eugenol, eucalyptol, myrtol, benzol, and several others may also be used. Liquid vaseline is a non-coagulant. It is usually prepared with a trace of benzoic acid. As vaseline by permeating the dentine makes it thereafter less permeable to water, an excellent dressing is found if you mix liquid vaseline with any of the oils named, or, better, with eugenol.

The coagulants which are frequently used in teeth, and yet should not be so used, are alcohol—how frequently men use this to dry a canal!—carbolic acid, camphophenique, aromatic sulp. acid, tinct. iodine, tinct. myrrh, tinct. opium, creosote, tannic acid, spirits camphor, hydronapthol, listerine, wine of opium, salicylic acid, and others. None of these should be used in a tooth root except as already advised. I have not mentioned iodoform. If it is an aqueous solution it is slightly a coagulant; but if it is a saturated solution in ether, it may be used, and I have used it in many cases with excellent results.

It is plain from what I have said, that I consider immediate root-filling contra-indicated in these cases. When the soreness has passed away the canal is to be cleansed thoroughly, dried, and filled. The method which I use is as follows: Red gutta-percha is dissolved in chloroform, and in it is placed a piece of waxed floss silk, which is then removed and allowed to harden somewhat by the evaporation of the chloroform. This gives you what is practically a cone of gutta-percha which has silk in it. The solution is pumped into the canal, and the gutta-percha and silk packed in till the cavity is filled. The object is that, despite the utmost care, pericementitis may recur in such a tooth, and renewed treatment becomes necessary. It is, as you all know, a difficult matter to remove a root-filling from such a tooth. With the filling here described there is no difficulty; because if one end of the silk is caught in the tweezers, the whole filling may be readily removed. At the same time, should such a removal be not indicated, the silk may be left in, because it is thoroughly imbedded in the gutta-percha, and cannot be compared with a cotton filling. This method is original with myself, but recently I have seen somewhere a similar suggestion, the gentleman using a twist of cotton, which is plainly not so good since the silk is stronger, more uniform in diameter, and may be prepared fine enough for very small canals. After a tooth of this kind has been brought to a comfortable stage, it should be filled with gutta-percha or oxy-phosphate for several months, rather than risk renewed disturbance by malleting gold.

In cases where what is known as "blind abscess" occurs, the treatment is similar, especially where there is a pus discharge through the root. That is to say, coagulants must not be used, and immediate root-filling must not be resorted to, nor a permanent filling inserted, till after several weeks or months. If, however, the operator should decide from the exigencies of the case to drill through the gum and process, thus producing artificially a fistula, the procedure thereafter would be the same as in ordinary abscess which presents with fistula.

The third condition is that of alveolar abscess proper, and is to be divided into two classes, acute and chronic, each with a special treatment.

The acute abscess presents with a puff of the gum tissue, and we open it with a bistoury, releasing the pus and relieving the pain. A tampon dipped in oil of cloves should be left in the opening, and the patient dismissed till the following day, nothing being done to the tooth. On the second day the tampon must be removed the sac thoroughly cleansed of pus, and if possible the diseased tissue burred from the end of the root with an engine burr. The canal may be attended to at this sitting if the soreness has subsided; if not, it should be postponed till the following day. All remains of the pulp and septic matter should be removed from the tooth, as well as all decay. The canal should be freely opened and reamed out. In cases of abscess, whether acute or chronic, the reamer is specially indicated, because the walls of the canal are saturated with septic matter and frequently softened to a cheesy consistency. All of this softened dentine must be removed, even though the canal be enlarged beyond the necessity for filling. To those who are horrified at the bare suggestion of reaming a canal, I would like to say that a root of a tooth is the same and requires the same treatment whether it has lost the crown or not; and I would remind these gentlemen that where the crown had been lost they do not hesitate to enlarge the canal for the reception of the post pin or screw used in fastening the crown into place. The root being thoroughly cleansed and all septic matter and softened dentine being removed, the procedure is different from that in either of the cases already described. In the first instance it was shown that the contents of the tubuli demanded our attention. They do also after abscess, though in a different way. It is needful that they be asepticized. Of course in all these operations it is understood that the rubber dam is in position, if at all practicable. The Evans root-dryer is to be used till there is a distinct feeling of heat on the part of the patient, and the hissing sound emitted from the contact of the heated point with the moisture present ceases entirely. As much moisture as possible being thus abstracted, one of the essential oils, let us say oil of cloves, is to be pumped in. The tooth thus dried out will absorb a great deal of the agent. This may be left for a minute or two, when the root is to be again dried out as before, and a second application of the oil made. This may be repeated three or four times, or until the operator feels assured that the agent has entered the tubuli thoroughly.

In these cases immediate root-filling is the only treatment which promises no return of the abscess. My practice is to pack at the extremity a small quantity of the canal paste recommended by Dr. Van Woert (oxide of zinc, iodol, and carbolized vaseline.) Right here it must be observed that, though this contains ingredients which are coagulants, they are not used in the tooth till the contents of the tubuli have been rendered aseptic. The main objection to coagulants is that, by coagulating the albumen in the contents of the tubuli at their ends, the openings are thus forever sealed against the entrance of antiseptics. Besides, combined in this paste they can be carried to the extremity of the root, which, remember, has been enlarged throughout its length without coming in more than temporary contact, if at all, with the sides. If coagulation occurs at the extremity, all the better, because the foramen is thus sealed up against the entrance of the dreaded bacteria. The canal is then sealed, as already described, with thin oxy-phosphate and a cone of gutta-percha.

In chronic abscess the procedure just described may be essayed first and the root and tooth filled. Should the fistula reappear, thus proving that the abscess persists, it becomes necessary to either operate or abandon the case as incurable. If an operation is decided on, as it probably would be with any of the anterior teeth, the patient should be anæsthetized. Those who have had success with cocaine may use that uncertain drug. I have never been attracted by its effects; and I am informed by some eminent specialist on the eye that there is a feeling now prevailing that, though cocaine may effect anæsthesia during the operation, it is noticed that healing afterward is neither so rapid nor so satisfactory as where it is not used. In some cases gas will serve your purpose. But in a cuspid, or a two rooted bicuspid, ether is the only satisfactory anæsthetic, because of the time involved in the operation. The patient under its influence, a quarter or even a third of the root or roots are to be amputated and removed. This is done as follows: with a spear-shaped drill, drill through the centre of the root from the labial toward the palatal side. Follow with a new sharp fissure burr operating laterally both ways, thus severing the end of the root. This may be done with little injury to the gum, and is not at all difficult. To remove the root after it has been amputated is the part requiring the anæsthetic, for even a skilled operator may take an hour to accomplish it. Sometimes it may be pried out at the first attempt, but in extreme cases the alveolar plate must be removed before the root can be extracted. This may involve a large opening in the gum, but with proper care it will result in a perfect cure. The filling of the root where amputation has been decided on at the outset, occurs after amputation, and may best be done on the day after. The wound should be packed in the interim with cotton. When the patient presents, the end of the root will be in view. I ream through the canal till I see the reamer emerge at this point; I smear the canal with solution of gutta-percha, and fill with a cone, forcing it through the end. Then with a burnisher make this smooth by removing the excess.

As a dressing for these cases and any similar wounds where a loss of tissue is to be restored by granulation, I have had the utmost satisfaction from liquid vaseline, which I have said contains benzoic acid, mixed with menthol, five per cent. This dressing will be found to be not only an antiseptic, but to a great extent sedative, leaving the patient in a most comfortable condition.

The fourth class of cases are those where crowns are to be used. Unfortunately these too seldom receive the treatment which would be accorded were the natural crowns in place. They are to be treated exactly as they would be if they were simply decayed teeth; and the crowning operation is to be decided on exactly as has been indicated for immediate root-filling. Wherever immediate root filling is indicated, the immediate setting of a crown after the other treatment has been attended to is permissible. Where immediate root-filling is contra-indicated, as in pericementitis and blind abscess, the crown should not be set until the tooth has been treated thoroughly, and then should be fastened with gutta-percha rather than with cement, just as a temporary filling was advised instead of a permanent one. To remove a crown set with gutta-percha, it is only necessary to heat the beaks of a pair of forceps, and hold the tooth till the heat has been transmitted, when the gutta-percha will become softened and the crown slip off easily.

In conclusion, I have to say that these directions, of course, do not apply to all cases; we can make no binding rules. In the matter of root-filling, a dentist must be a physician; he must recognize the pathological condition with which he is dealing, and be governed accordingly. His mechanical skill will be needed to perfectly carry out his method; but the most skilful mechanical dentist will not produce good results unaided by a knowledge of the symptoms of health and disease.

Again, I am aware that these methods cannot be applied in all teeth, even where they may be indicated. The buccal roots of superior molars and the anterior roots of lower molars often tax our utmost patience. The man who claims to thoroughly fill all such roots perverts the facts in the case, and imagines himself more skillful than it is possible for any man to be or to become. I have teeth in my possession which I will defy any man to fill from foramen to crown, with the whole operation in view, and appreciation of the conditions more possible than it could be were such a tooth in the mouth. What shall we do in this case? Do our best, since no man may do more. But our best involves a great deal more work than many give to these cases. It is true that many roots cannot be filled, but it is also true that many roots are left empty because the operator was either unable or unwilling to fill them. A tooth with a pulpless, unfilled root is like an edifice with a defective foundation. Sooner than abandon such a root, I would cut away half the crown to gain access. It would be even better to cut the whole crown off and supply a substitute, if the root or roots could not otherwise be thoroughly filled.

Let us be thorough in all our work; but above all let us spare no pains to be thorough in filling roots.—*Dental Mirror.*

---

### Communications.

### AMERICAN DENTAL ASSOCIATION.

*Editor of the American Journal of Dental Science:*

The thirtieth annual session of the American Dental Association, will be held at Excelsior Springs, Missouri, commencing Tuesday, August 5th, 1890, at 10 o'clock, A. M.

GEO. H. FUSHING,
Recording Secretary.

---

### NATIONAL ASSOCIATION OF DENTAL EXAMINERS

*Editor of the American Journal of Dental Science:*

